# Proteins and Protein Metabolism

**Published:** 1906-12

**Authors:** J. M. Fortescue-Brickdale

**Affiliations:** Medical Registrar, Bristol Royal Infirmary; Senior Physician to Out-Patients, Royal Hospital for Sick Children and Women; Demonstrator of Physiology, University College, Bristol


### PROTEINS AND PROTEIN METABOLISM.

BY

J. M. FORTESCUE-BRICKDALE, M.A., M.D., Oxon.,

*Medical Registrar, Bristol Royal Infirmary; Senior Physician to Out-Patients, Royal Hospital for Sick Children and Women; Demonstrator of Physiology, University College, Bristol.*

---

In this article it is proposed to deal as briefly as possible firstly with some recent views on the chemical structure of the protein molecule, and secondly with a few of the changes which proteins undergo in the body during digestion, absorption, and elimination.

*The Chemistry of Protein.*—The large group to which the term protein is applied consists of a number of more or less closely-related substances, which have been arranged in sub-groups according to the complexity of their structure. The simplest of these are the protamines and histones, the more complex the "albugins"[1] (albumins and globulins), and the most complex the conjugated proteins or proteides, in which additional groups are added to the protein molecule.

The construction of the protein molecule may conceivably be demonstrated either by analysis or by synthesis. At present, owing to the peculiar difficulties which surround the task, neither of these methods has been completely successful. It has, however, been possible to split off from the proteins certain groups of atoms the chemical structure of which can be determined; and by way of synthesis, Emil Fischer has recently succeeded in piecing together a number of these atom groups to form a substance which closely resembles proteose, a simpler protein naturally derived by hydrolysis from the more complicated molecules during the process known as digestion. A class of substances which has been found to constitute a great number of the atom groups which can be split off from protein is that known as the amino-acids. These

[1] This name has been officially dropped "like a hot potato," but no cooler substitute has been offered in its place.

are bodies in which the hydroxyl group of an oxy-acid is replaced by $NH_2$; thus

Glycocoll $CH_2NH_2COOH$

may be regarded as a derivation of

Oxy-acetic acid $CH_2OHCOOH$.

A large number of these amino-acids exist: some are mono-amino acids, *i.e.* they contain only one amine or $NH_2$ group; others are diamino acids, and contain two. They may also contain an aromatic ring, such as tyrosin, which is para-oxy-phenyl-amino-propionic acid.

$$C_6H_4,OH,CH_2CHNH_2COOH.$$

$$1:4.$$

The simplest proteins consist of these amino-acids only: the protamines, for instance, consist as to 80 per cent. of an amino-acid called "arginine," the rest being leucin or amino-caproic acid. Arginine is what is known as a binary product, that is, it can be further split up into two others, though this is not accomplished by hydrolysis. The two substances are ornithin, an amino-acid, and guanidine, the former constituting as it were the bulk and the latter a sort of side chain in the molecule. Guanidine has the formula

$$\begin{array}{c} NH_2 \\ | \\ C{=}NH \\ | \\ NH_2 \end{array}$$

and it is probably the number of free guanidine side chains bearing the basic group $NH_2$ at their extremities which gives to protamine its basic character. It will be noted that guanidine is closely related to urea or carbamide.

It has been estimated that protein may consist of about 100 to 120 atom groups or "nuclei"; the simple proteins do not differ from the more complicated ones in the number of nuclei they contain, but merely in showing less variety in the composition of the groups.

Hæmoglobin (a compound of hæmatin and a histone globin), being crystallisable, has had its molecular weight estimated. 100 grams Hb absorb ·2264 grams of oxygen; this would give 14,250 as the molecular weight. The iron content brings out the molecular weight at about 16,000, and other methods give closely similar results. Hæmatin itself has a molecular weight of about 600, and if this be deducted, globin will have a molecular weight of about 14,400. The average molecular weight of the known "nuclei" or radicles is about 140. When these unite together the condensation is accompanied by the loss of one molecule of water, so that 18, its molecular weight, must therefore be deducted, and about 120 put down as the molecular weight of an atom group in composition. For globin, therefore, $14{,}400 \div 120 = 120$ will be the number of atom groups in the molecule.

To the amino-acid groups Fischer has given the name "peptide"; a combination of two of these would be a "dipeptide," and of several a "polypeptide." These polypeptides are the component groups of proteoses. With certain exceptions, one of which has been noted above, proteins all have an amphoteric reaction, that is they are weakly acid and weakly basic at the same time. That they are not strongly one or the other is due to the way in which the amino-acids combine. Supposing two amino-acids—$R_1CHNH_2COOH$ and $R_2CHNH_2COOH$—are to be combined, where $R_1$ and $R_2$ represent any radicles. These can be written—

$$\begin{array}{l} H_2N,\underset{\displaystyle R_1}{\underset{|}{CH}},CO[OHH]NH,\underset{\displaystyle R_2}{\underset{|}{C}}HCOOH \end{array}$$

If the OH and H enclosed in brackets go out as water, the resulting substance will have at one end a basic $H_2N$ group and at the other an acid COOH group, and thus the amphoteric character will be produced. Decomposition will, moreover, be possible by hydrolysis, *i.e.* by absorption of the OHH group eliminated in the formation of the compound.

Oxidation of a polypeptide will first affect the side chains, represented by $R_1$ and $R_2$ in the formula above. These eventually may be oxidised to COOH, and we shall then have—

$$H_2N,\underset{\displaystyle COOH}{\underset{|}{CH}},CO,NH,\underset{\displaystyle COOH}{\underset{|}{CH}},COOH$$

Further oxidation splits off $CO_2$ and turns the CH into CO groups—

$$H_2N,CO,CO,NH,CO,COOH.$$

This very unstable grouping would hydrolyse itself into

$CONH_2(COOH)$ or oxalamide, which then again is easily hydrolysed into oxalic acid, $COOH,COOH$ and ammonia $NH_3$; in fact, these are the principal products when protein is oxidised by potassium permanganate.

Further changes produced by the agency of bacteria may take place in the cleavage products of protein. Thus arginine may be oxidised to putrescine—

$$CH_2NH_2(CH_2)_2CH_2NH_2$$

and from lysin

$$CH_2NH_2(CH_2)_3CHNH_2COOH.$$

cadaverine is formed

$$CH_2NH_2(CH_2)_3CH_2NH_2$$

$CO_2$ being split off.

These substances, the products of decomposition of animal bodies, are with their oxidised products known as "ptomaines."

Less is known of the atom groups not amino-acid in structure. Some, at any rate in the case of several protein substances, are of the nature of carbohydrates. A large amount of physiological and pathological evidence is now available which shows, both from the analytic and synthetic side, that a carbohydrate nucleus can be built into the protein molecule. Pavy has succeeded in splitting off a carbohydrate from many protein bodies, and regards this fact as of fundamental importance to a right conception of the nature of diabetes mellitus.

Glycosamine is the best-known of these carbohydrate nuclei.

$$CHO,CHNH_2(CHOH)_3CH_2OH.$$

It probably exists in a polymeric form in the protein molecule, which may be split up by hydrolysis.

Glucoproteides form a special class of proteins, in which the carbohydrate portion can be split off without destroying the proteid character of the molecule.

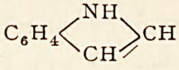

The aromatic nuclei are of especial interest in connection with the excretion of indol and skatol. These toxic substances are in the liver oxidised and combined as sulphates, in which form they appear in the urine; a failure in this process may account for some auto-intoxications. Indol, which has the formula and skatol, which is methyl indol, do not exist as such in protein. In their place is tryptophane—

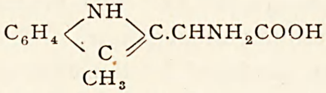

which gives rise to the Adamkievicz reaction characteristic of protein substances.

Phenylalanine and tyrosine, other aromatic nuclei, are precursors of homogentisic acid, a body which reduces Fehling's solution. It is chemically dioxyphenyl-acetic acid $C_6H_3OH$, $OH,CH_2COOH$ (alkaptonuria). Of the sulphur-containing groups cystin is the most important. Cystin is a "binary compound," but it cannot, like arginine, be split up by hydrolysis. It is formed from two molecules of cysteine and amino $\beta$ thiopropionic acid, with the loss of a molecule of hydrogen, not of water.

Cystin is $CH_2SH,CHNH_2COOH$, and is thus closely related to taurine—$CH_2SOOH,CH_2NH_2$; in fact, cysteine can be converted into taurine in the laboratory.

*Protein Metabolism.*—Considerable changes have recently taken place in current opinion on this subject. Not only have new ferments acting on the foodstuffs been demonstrated and an entirely new set of bodies called "hormones" been described, of which the duodenal "secretin" is a type, but the katabolic processes through which protein bodies normally pass have been shown to be much more extensive than was formerly imagined. The intermediate products of protein katabolism are still but imperfectly known; it seems clear, however, that while certain molecules are passing through the various disintegration stages of proteose (albumose) and peptone (polypeptide), others are split up directly into their simple end products—the amino acids. In the same way the amino-acids are also constantly being split off from the albumoses and peptones. There would appear, therefore, to be various paths for protein katabolism. The molecule may either take the high road via primary albumose, secondary albumose, peptone (or polypeptide), or may take a short cut at any point on the route and arrive at once at its amino-acid termination. It [illegible]as been observed that the tryptic and intestinal digestion is the one which carries protein to its final stage, gastric digestion going no further than the production of polypeptides. Food, however, which has been acted on by the gastric secretion, is more completely and surely attacked later on in the small intestine, so that surgeons should not too hastily conclude that the stomach is a mere developmental relic, and of no value to the organism.

The primary albumoses (hetero- and proto-albumose) differ in the amino-acids produced by their hydrolysis, hetero-albumose giving rise to much tyrosin and glycocoll, proto-albumose to much leucin. The primary albumoses never give rise to a carbohydrate group, which, however, is found in a form of secondary albumose known as syn-albumose, which is therefore supposed to be split directly from the protein molecule.

The final cleavage products of protein have been shewn in dogs to be capable of supporting life and maintaining the nitrogenous equilibrium of the animal.

The amino-acids are probably carried to the liver by the portal blood, but technical difficulties have hitherto prevented the complete proof of this. Ammonia, a still more simple cleavage product, has, however, been demonstrated to exist in excess in portal blood. Some portion of the amino-acids are probably resynthesised into tissue protein in the liver, but the greater part is apparently there converted into urea and excreted by the kidneys. A special ferment, "arginase," has been isolated from the liver which can decompose arginine into urea and ornithine.

Urea may be regarded as derived from protein in three ways. The principal precursor is glycocoll, $CH_2NH_2COOH$, which, though not found to any extent in true proteins, is abundant in sclero-proteins (albuminoids) such as gelatine. The second way in which urea can be found is from the guanidine group, which we have seen occurring in arginine. Thirdly, it may result from the oxidation of uric acid in the liver and tissues. Uric acid itself is an oxidation product, being tri-oxypurin. The purin bases from which it is formed are derivatives of the nucleo-proteides of all nuclei (the chromatin substance of the histologist), through the intermediate stages of nuclein and nucleic acid.

The purin bases are a group of bodies derived, chemically speaking, from purin, a substance which does not occur in the animal body. It is a double ring compound—

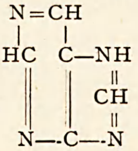

and from it oxidation compounds and substitution products can be formed. The best known is tri-oxypurin, or uric acid—

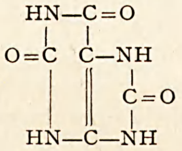

If the two side columns are split off, and additional hydrogen absorbed to saturate the nitrogen (triad)—

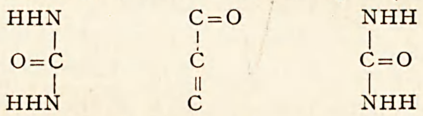

two molecules of urea are produced.

It seems probable, however, that the uric acid found in mammalian urine is mainly derived from nucleo-proteides, and does not represent an incomplete stage of urea formation from protein. In birds, where uric acid is the main nitrogenous excretion product, its formation is probably not on analogous lines, but due to a special synthetic process.

The amino-purins, adenine and guanine—

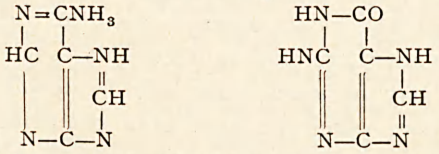

have been held to be the toxic intermediary products responsible for the symptoms of goutiness. The oxidation of purin bases into uric acid is probably the work of a special ferment, and the absence of this ferment may be the most important pathogenic factor in that condition.

